# The evaluation of skin sensitization potential of the UVCB substance diisopentyl phthalate by in silico and in vitro methods

**DOI:** 10.1007/s00204-024-03738-x

**Published:** 2024-05-28

**Authors:** Isisdoris Rodrigues de Souza, Martina Iulini, Valentina Galbiati, Ana Carolina Rodrigues, Daniela Fiori Gradia, Anderson J. M. Andrade, James W. Firman, Cynthia Pestana, Daniela Morais Leme, Emanuela Corsini

**Affiliations:** 1 Graduate Program in Genetics, Department of Genetics, Federal University of Paraná (UFPR), Curitiba, PR Brazil; 2 Department of Physiology, Federal University of Paraná (UFPR), Curitiba, PR Brazil; 3 Laboratory of Toxicology, Department of Pharmacological and Biomolecular Sciences ‘Rodolfo Paoletti’, Università Degli Studi di Milano, Via Balzaretti 9, 20133 Milan, Italy; 4 National Institute for Alternative Technologies of Detection, Toxicological Evaluation and Removal of Micropollutants and Radioactives (INCT-DATREM), Institute of Chemistry, Araraquara, SP Brazil; 5 School of Pharmacy and Biomolecular Sciences, Liverpool John Moores University, Liverpool, UK

**Keywords:** New approach methodologies, QSAR models, Keratinocytes, THP-1 activation assay, Long non-coding RNAs

## Abstract

Diisopentyl phthalate (DiPeP) is primarily used as a plasticizer or additive within the production of polyvinyl chloride (PVC), and has many additional industrial applications. Its metabolites were recently found in urinary samples of pregnant women; thus, this substance is of concern as relates to human exposure. Depending upon the nature of the alcohol used in its synthesis, DiPeP may exist either as a mixture consisting of several branched positional isomers, or as a single defined structure. This article investigates the skin sensitization potential and immunomodulatory effects of DiPeP CAS No. 84777-06-0, which is currently marketed and classified as a UVCB substance, by in silico and in vitro methods. Our findings showed an immunomodulatory effect for DiPeP in LPS-induced THP-1 activation assay (increased CD54 expression). In silico predictions using QSAR TOOLBOX 4.5, ToxTree, and VEGA did not identify DiPeP, in the form of a discrete compound, as a skin sensitizer. The keratinocyte activation (Key Event 2 (KE2) of the adverse outcome pathway (AOP) for skin sensitization) was evaluated by two different test methods (HaCaT assay and RHE assay), and results were discordant. While the HaCaT assay showed that DiPeP can activate keratinocytes (increased levels of IL-6, IL-8, IL-1α, and *ILA* gene expression), in the RHE assay, DiPeP slightly increased IL-6 release. Although inconclusive for KE2, the role of DiPeP in KE3 (dendritic cell activation) was demonstrated by the increased levels of CD54 and IL-8 and TNF-α in THP-1 cells (THP-1 activation assay). Altogether, findings were inconclusive regarding the skin sensitization potential of the UVCB DiPeP—disagreeing with the results of DiPeP in the form of discrete compound (skin sensitizer by the LLNA assay). Additional studies are needed to elucidate the differences between DiPeP isomer forms, and to better understand the applicability domains of non-animal methods in identifying skin sensitization hazards of UVCB substances.

**Supplementary Information:**

The online version contains supplementary material available at 10.1007/s00204-024-03738-x.

## Introduction

Assessing the potential risks posed by substances of unknown or variable composition, complex reaction products, or biological materials (UVCBs) presents a particular challenge to regulatory agencies (Salvito et al. 2020). These substances can contain a large number of constituents, and their composition can be variable, depending upon their source materials and manufacturing processes. In addition, it may be technically challenging to identify and test the toxicity of each individual constituent present in a UVCB, and, hence, to conduct risk assessments, determining appropriate classification and labeling needs (Salvito et al. 2020).

Diisopentyl phthalate (DiPeP) belongs to the group of low-molecular weight phthalate esters. It bears a pair of saturated alkyl substituents, each consisting of five carbon atoms (a butyl chain with branching methyl unit). Although its use in cosmetics and personal care products is banned in several jurisdictions, such as Europe (ECHA 2022a, b) and Brazil (ANVISA 2016), the substance is still industrially produced application as a plasticizer. It is commonly found in ammunition propellants and products such as shoes and hoses (*e.g.*, garden hoses) (Petrom 2016).

DiPeP (registered under CAS No. 605-50-5) is available in the form of a discrete compound (Table 1, ID 1) and is a potential skin sensitizer according to the mouse local lymph node assay (LLNA)—although not a skin irritant (ECHA 2023). It is also toxic for reproduction (category 1B) (ECHA 2023). In Brazil, the principal marketed formulation of DiPeP (assigned CAS No. 84777-06-0) instead represents a mixture consisting of both Table 1, ID 1 (85%) and its positional isomer Table 1, ID 2 (15%). This feature arises because of its dominant means of production—the esterification of phthalic anhydride with the corresponding mixture of isomeric isoamyl alcohols (Bertoncello Souza et al. 2018). ECHA describes DiPeP under CAS No. 84777-06-0 as a UVCB. This substance has greater potency than other phthalate esters in inhibiting rat fetal testicular testosterone production, and its metabolites have further been detected in urine samples of Brazilian children and pregnant women through biomonitoring studies (Rocha et al. 2017; Bertoncello Souza et al. 2018). Of note, different phthalate structures reveal varying absorption levels and accumulation properties in the skin, and present different trends in altering skin protein expression and inflammation after exposure (Pan et al. 2014; Sugino et al. 2017).

**Table 1:** Structures and identifiers relating to DiPeP mixture components, alongside the products of their esterase-mediated primary metabolism

ID	Name	Structure	CAS	Chemical form	Status
–	Diisopentyl phthalate (mixture)	See below	84777-06-0	Phthalate diester	Composition: Substance 1 (85%) Substance 2 (15%)
1	Bis(3-methylbutyl) phthalate		605-50-5	Phthalate diester	Major mixture component: May be marketed alone as diisopentyl phthalate
2	Di(2-methylbutyl) phthalate		68951-39-3	Phthalate diester	Minor mixture component
3	Monoisopentyl phthalate		17866-76-1	Phthalate monoester	Putative primary metabolite of 1
4	Isopentanol		123-51-3	Alkyl alcohol	Putative primary metabolite of 1
5	Mono(2-methylbutyl) phthalate		–	Phthalate monoester	Putative primary metabolite of 2
6	2-Methyl-1-butanol		137-32-6	Alkyl alcohol	Putative primary metabolite of 2

Skin sensitization is a process by which a substance induces allergic response following repeated skin contact. Its key biological events are well known, such that an adverse outcome pathway (AOP) describing the endpoint has been formally developed (Sakuratani et al. 2018). This consists of a sequence of four key events (KE): KE1, covalent binding of haptens to nucleophilic centers in skin proteins (the molecular initiating event, or MIE); KE2, keratinocyte activation, by inducing an inflammatory response and changing the expression of genes associated with specific cell signaling pathways; KE3, dendritic cell activation; and KE4, the proliferation of antigen-specific T cells (OECD 2021). There exists a consensus that the best approach for assessing skin sensitization potential lies in integration of multiple sources of information. Through weighing all relevant existing evidence, the targeted generation of new data, as needed, may be guided. With respect to this new data, information can be provided by a combination of non-animal methods (*e.g.*, in silico predictions, in chemico and in vitro assays), as described in the recently launched OECD Defined Approach (DA) guideline (OECD 2021). Additionally, other non-animal assessment strategies are currently being developed and validated, and more approaches may be adopted for regulatory purposes in the future (Kleinstreuer et al. 2018). Specifically to KE2, both HaCaT assay and reconstructed human epidermis (RHE) IL-18 assay have been proposed to evaluate keratinocyte activation alternatively to KeratinoSens™ and LuSens OECD assays (Jeon et al. 2019). The HaCaT assay, quantifying IL-1α and IL-6 cytokine increase, presented approximately 83% accuracy in identifying skin sensitizers (Jeon et al. 2019). RHE IL-18 assay uses in vitro epidermal tissues, presenting the ability to distinguish sensitizers from non-sensitizers with 95% accuracy (Gibbs et al. 2013). For dendritic cell activation, the h-CLAT assay provides the basis for quantifying two surface markers with roles in dendritic cell (DC) activation, using THP-1 as surrogate of DC. Although it is an adopted OECD test method (OECD TG442 E), scientific literature has shown h-CLAT demonstrates a failure rate of about 30% in hazard identification (Mitjans et al. 2008), and that the association of this method with quantification of IL-8 increases sensitivity in identifying allergens (Mitjans et al. 2008, 2010). Thus, the combination of the h-CLAT with IL-8 quantification has been referred as THP-1 activation assay (Iulini et al. 2022).

Considering that skin is subjected to be exposed simultaneously to a variety of molecules, risk of exposure to chemicals also includes their capacity to immunomodulate skin responses to other chemicals (Nowak et al. 2019).

Chemicals with the potential to present immunomodulatory effects can enhance inflammatory reactions on the skin, affect the development of allergy and, consequently, affect human health in ways not anticipated in the assessment of the chemical individually (Corsini et al. 2011, 2012; de Souza et al. 2023; Wong Lau et al. 2023). Along these lines, LPS-induced THP-1 activation assay has been proposed to address immunomodulatory effects of chemicals and it is based on the observation of an increase or a decrease in the response of cells to LPS, after exposure to the tested substances, indicating enhancement or suppression of immune response (DC activation) to an inflammatory agent (Bosshart and Heinzelmann 2016; Galbiati et al. 2010; Masi et al. 2022). Additionally, long non-coding RNAs (lncRNA) have been associated with chronic inflammatory skin diseases and have been shown to be potential biomarkers in epidermal homeostasis (Mervis and McGee 2020; Wang et al. 2018; Shefler et al. 2022). Specifically, lncRNA genes *MALAT1* and *NEAT1* are highly expressed in keratinocytes and can be taken as signaling mediators of cytokine-dependent pathways (Zhang et al. 2021; Zhao et al. 2016; Zhu et al. 2021; Shefler et al. 2022).

Whole-mixture testing is considered the best approach for the toxicity assessment of UVCB substances. If the identities of the constituents are known, then grouping, read across, and mixture toxicity modeling represent complementary approaches to address data gaps (Lai et al. 2022). Considering that the discrete DiPeP compound is classified as a potential skin sensitizer according to experimental results (LLNA), and that DiPeP under CAS No. 84777-06-0 is described as a UVCB, the effects and limitations of using new approach methodologies (NAMs) in characterizing the hazard potential of this latter “difficult-to-test substance” were evaluated using an assortment of complementary in silico and in vitro methods (both OECD-approved and non-conventional). The same set of approaches was successfully applied in a previous study relating to 4-Octylphenol (OP), an environmental contaminant with widespread distribution (de Souza et al. 2023).

## Materials and methods

### Tested substances

Diisopentyl phthalate (DiPeP; CAS No. 84777-06-0), provided by PETROM (Petroquímica Mogi das Cruzes—Mogi das Cruzes, SP, Brazil) with a purity of 99%, was used for the in vitro experiments described in Sect. “In vitro assays”.

**Fig. 1:**
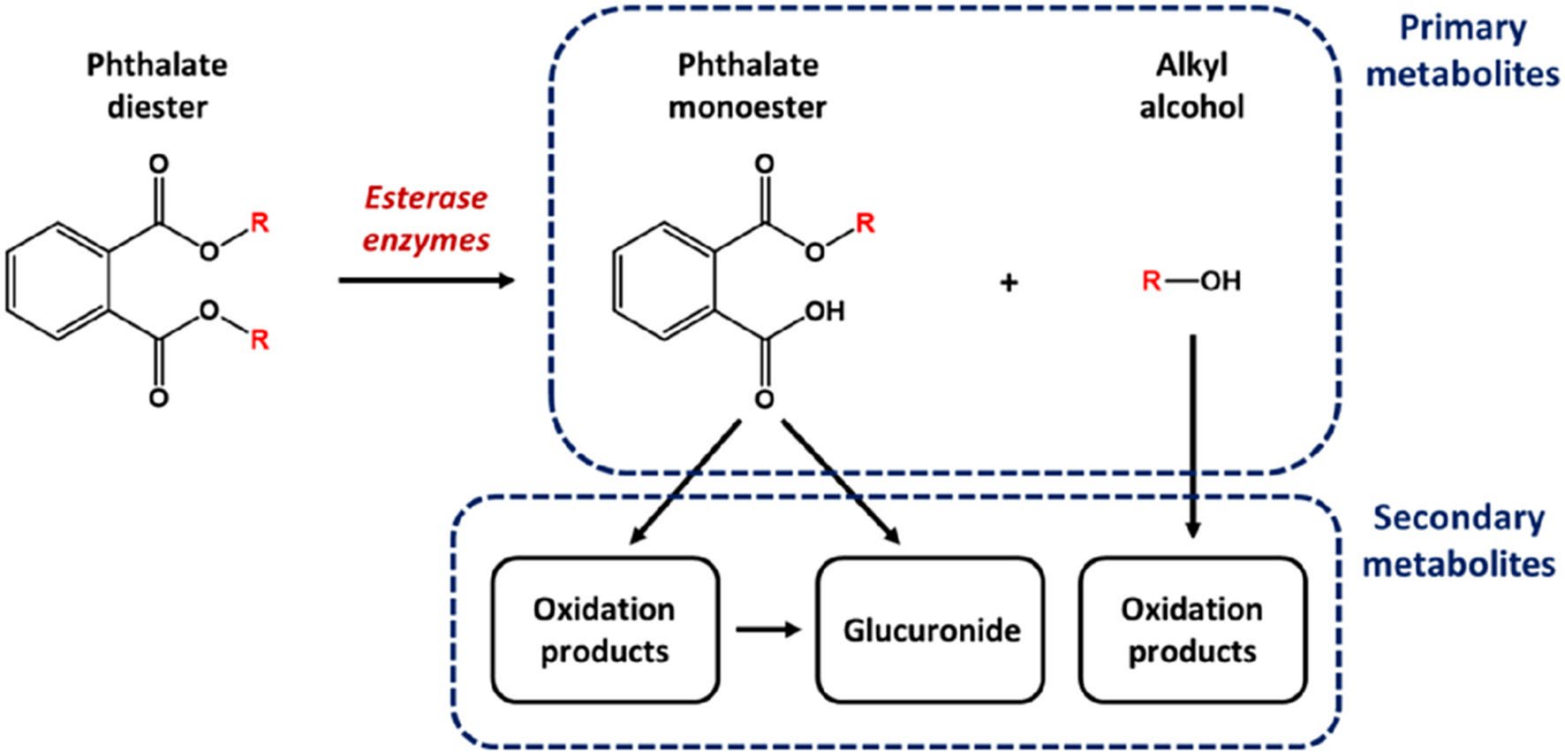
Overview of generic phthalate ester metabolic pathways, indicating identities of “primary” and “secondary” metabolites

As DiPeP represents a mixture of phthalate esters, in silico predictions were conducted using its main components. These appear within Table 1—listed, respectively, under IDs 1 (accounting for 85% of commercially-supplied mixture) and 2 (the remaining 15%). Possible metabolites were also investigated, considering the typical metabolism of a phthalate diester (as explained within Fig. 1). This metabolic pathway proceeds initially through esterase-mediated hydrolysis of the parent diester (Hopf et al. 2014; Frederiksen et al. 2007), yielding a monoester derivative, alongside the cleaved alkyl alcohol, as “primary metabolites” (each additionally represented within Table 1, under IDs 3, 4, 5, and 6). Further modification to these structures is acknowledged to occur, leading to the formation of “secondary metabolites”. Such transformations include glucuronic acid conjugation at the free carboxyl function of the monoester—or else a series of progressive oxidations at the remaining alkyl substituent. The free alcohol is likewise liable to experience oxidative modification, ultimately undergoing conversion into a carboxylic acid form (Lachenmeier 2008). Products beyond primary metabolites were not considered within our assessment.

### In silico data

Each of the six substances depicted within Table 1 were subjected to in silico examination. A selection of seven tools relating to skin sensitization were used for their profiling and prediction. These included OECD QSAR Toolbox structural alert-based profilers (version 4.5 at www.qsartoolbox.org) related to skin sensitization and general protein binding, the Toxtree v.2.6.13 skin sensitization reactivity domains based on the identification of mechanisms of action for skin sensitization (Enoch et al. 2008), and two statistical QSAR models retrieved from VEGA (Evaluation of chemicals within a Global Architecture; version 1.2.0; available at www.vegahub.eu) based on the LLNA. Each of four KE stages within the corresponding AOP were described by at least one of these tools (please refer to Table 2, and the appropriate referenced software documentation, for additional information on all).

**Table 2:** Details relating to identity, described endpoint, and accessibility of in silico tools employed within this study. Please refer to appropriate documentation (issued alongside software) for additional information

Form	Profiler/model title	Endpoint described	Aligned KE	Software details			
				Program	Version	Accessibility	Ref
Structural profilers	Skin sensitization reactivity domains	Molecular fragments mechanistically associated with holding potential to reactively modify skin proteins (thus inducing hapten formation)	KE1	Toxtree	3.1.0	toxtree.sourceforge.net	Patlewicz et al. (2008)
	Protein binding alerts for skin sensitization according to GHS		KE1	OECD QSAR toolbox	4.5	qsartoolbox.org	Schultz et al. (2018)
	Protein binding alerts for skin sensitization by OASIS						
	Keratinocyte gene expression	Data derived from KeratinoSens assay, which examines stimulation of antioxidant response in immortalized human keratinocytes	KE2				
	Protein binding potency h-CLAT	Data derived from h-CLAT assay, which quantifies changes in expression of cell surface markers (i.e., CD86 and CD54)	KE3				
Statistical model/QSAR	Skin Sensitization (CAESAR)	Data derived from mouse LLNA, which evaluates proliferation of lymphocytes	KE4	VEGA QSAR	1.1.5	vegahub.eu	Benfenati and Manganaro (2013)
	Skin sensitization (IRFMN/JRC)						

The OECD QSAR Toolbox skin metabolism simulator was applied to parent compounds (ID 1 and 2). This tool mimics biotransformation of chemicals within the skin compartment, based upon assumption that enzymes responsible for xenobiotic metabolism within the liver are likewise expressed at that site (Mekenyan et al. 2012). However, only pathways leading to known primary metabolites 3, 4, 5, and 6 were found to be included within.

### In vitro assays

#### In vitro models

Three in vitro models were used in this study: HaCaT cells (immortalized human keratinocyte cell line, Rio de Janeiro Cell Bank—BCRJ, Cat. No. 0341, Brazil); THP-1 cells (human leukemia monocytic *cell line,* Elabscience Biotechnology Inc., Cat. No. EP-CL-0233, Houston, Texas, USA); and RHE model (EpiDerm™, MatTek Corporation, Bratislava, Slovakia). Culture procedures for these in vitro models are briefly described below.

HaCaT cells were cultured in Dulbecco’s Modified Eagle Medium (DMEM) supplemented with 10% heat-inactivated fetal bovine serum (FBS) (both from Gibco, Life Technologies, USA), 100 IU/mL penicillin G, 100 mg/mL streptomycin, and 1 µg/mL amphotericin. THP-1 cells were cultured in Roswell Park Memorial Institute (RPMI)-1640 medium (Sigma-Aldrich) supplemented with 2 mM L-glutamine, 50 μM 2-mercaptoethanol, 10% heat-inactivated FBS, 100 IU/mL penicillin, 100 μg/mL streptomycin, and 0.01 ng/mL gentamycin. Both cell cultures were maintained at 37 °C, in an atmosphere of 5% CO_2_ and with 95% relative humidity. Subcultures were performed either when cells reached approximately 80% confluency (HaCaT), or every 3–4 days (THP-1). RHE models were maintained according to the manufacturer’s instructions (EpiDerm™, MatTek Corporation) in the DMEM-based tissue culture medium provided, at 37 °C, with 5% CO_2,_ and at 95% relative humidity for 24 h (stabilization period before performing the experiments).

#### Test concentrations and exposure conditions

HaCaT and THP-1 cells were exposed to DiPeP at 0.03, 0.3, 3, 30, and 300 nM (sub-cytotoxic concentrations defined by cell viability assays) for all biomarkers evaluated, with the exception of gene expression (RT-qPCR), for which change in the expression of cytokines and lncRNA genes was evaluated only at 300 nM DiPeP. Dimethyl sulfoxide (DMSO) was used as a vehicle to prepare the stock solution of DiPeP, with 0.1%-v/v DMSO considered as the final concentration in the exposure media as well as in vehicle control. Exposure of 2D cell culture systems was carried out directly in the culture medium for 24 h, except to evaluate potential immunomodulatory effects, HLA-DR quantification and CD86. For that, THP-1 cells were first exposed to DiPeP for 24 h, before DiPeP + LPS was added for another 24-h treatment. In evaluating HLA-DR expression, THP-1 cells were exposed to DiPeP for 72 h, since HLA-DR may require longer periods of exposure to chemicals to be expressed at a detectable level (Iulini et al. 2022). CD86 quantification was performed following 24 h and 72 h of DiPeP exposure, in order to understand underlying mechanisms of its expression. All experiments were accomplished in the presence of negative control (cells cultured in medium only).

In the RHE model, the biomarkers were evaluated after 24-h treatment with 30 and 300 µM DiPeP prepared in acetone olive oil (AOO) (4:1) (solvent control). Topical exposure was used for this model; thus, Finn Chamber filter paper discs of 8 mm (SmartPractice, USA) were first impregnated with 25 µL of DiPeP solutions or AOO (vehicle control) and then placed on the top of the RHE model.

Positive controls (PC), wherein used, are outlined in their respective assay descriptions.

#### Cell viability assays

Cell viability assays were used to select sub-cytotoxic DiPeP concentrations, and were chosen according to those best indicated for each in vitro model. Thus, for keratinocytes-based models (HaCaT cell line and RHE tissues), the MTT assay (3-(4,5-dimethylthiazol-2-yl)-2,5-diphenyltetrazolium bromide, Sigma-Aldrich) was applied, while propidium iodide (PI, Sigma-Aldrich) in flow cytometry was used to evaluate THP-1 cells after DiPeP exposure.

In the MTT assay, HaCaT cells (5 × 10^4^ cells/well, 96-well plate) exposed to DiPeP were incubated with MTT at 0.5 mg/mL for 3 h, with formazan crystals produced dissolved in DMSO. Exposed RHE tissues were incubated with MTT at 5 mg/mL for 3 h, after which they were transferred to 24-well plates containing isopropanol. Plates were sealed with parafilm, incubated overnight at room temperature (RT) under orbital shaker, and protected from light to extract the formazan crystals. RHE tissues were discarded, and the extracted solutions were transferred to 96-well plate for absorbance readouts. In both keratinocyte-based models (HaCaT and RHE), absorbance values were obtained at 540 nm and 570 nm by the microplate reader Infinite 200™ (Tecan) (Mosmann 1983).

THP-1 cells (10^6^ cells/mL) exposed to DiPeP were stained with PI (0.625 μg/mL), and the fluorescence intensity of labeled cells was acquired by flow cytometer (NovoCyte 3000, ACEA Biosciences, Inc).

For cytotoxicity measured by MTT assay, the concentrations selected resulted in a of 80% viability in HaCaT cells. Viability of treated cells was calculated by comparing it with that of SC (DMSO), which was set as 100%. The viability of the vehicle control-treated cells (AOO 4:1) was set as 100%. For cytotoxicity measured by PI-stained THP-1 cells, the highest selected concentration of the tested chemical was that resulting in 75% viability (CV75).

#### ELISA: quantification of cytokines

Cytokines were quantified in HaCaT cells (IL-1α, IL-6, IL-8, IL-18, and IL-10) and RHE model (IL-1α, IL-6, IL-8, and IL-18) and THP-1 cells (IL-8, TNF-α, and IL-10). All cytokines evaluated in RHE and THP-1 cells were quantified in culture supernatants. However, in HaCaT cells, only IL-6 and IL-8 were evaluated in the culture supernatants, while IL-1α, IL-18 and IL-10 were examined in their intracellular content, since the release were not detected. For quantifying the intracellular cytokines, HaCaT cells (2 × 10^5^ cells/well, 24-well plate) were incubated in EDTA 0.05% v/v for 5 min at 37 °C in a 5% CO_2_ and air atmosphere, and then lysed in Triton-100 0.5%-v/v for 15 min on ice. Cell lysates were harvested and stored at −80 °C until analysis. Total protein was also determined by the bicinchoninic acid method (BCA), to normalize intracellular cytokine data (Corsini et al. 2013). 2,4-dinitrochlorobenzene (DNCB) 0.15%-v/v was used as PC in RHE models.

The following ELISA kits were used in the present study: Human IL-6 ImmunoTools sandwich ELISA (Cat. No. 31670069), Human IL-8 ImmunoTools sandwich ELISA (Cat. No. 31670089), Human IL-1α ELISA MAX^TM^ Deluxe Set (Cat. No. 445804), Human IL-18 ELISA kit MBL (Cat. No. 7620; Nagoya, Japan), DuoSet® ELISA Development System Human TNF-α (R&D Systems—Cat. No. DY210-05), and Human IL-10 ImmunoTools sandwich ELISA (Cat.n° 31670109U1). Cytokines were quantified in line the supplier instructions. The absorbance of the microplates was read at 450 nm. Experiments were carried out in three technical replicates and 3 biological replicates, except for the results obtained using the RHE model, for which two different tissue batches were used (*n* = 2).

For interleukin quantification by ELISA, the results were calculated in pg/mL from a standard curve to determine the Stimulation Index (SI). The SI was obtained by dividing the concentration of interleukin in pg/mL of treated samples with the concentration of interleukin in pg/mL of SC (fold-change). For intracellular interleukin quantification (IL-18 and IL-1α), results were expressed as pg/mg of total cell protein as assessed by the BCA protein determination method, as shown in the following equation:

$${\text{IL - 18}} = \frac{{{\text{IL - 18}}({\text{pg/mL}})\;{\text{in}}\;{\text{cell}}\;{\text{lysate}}}}{{{\text{Total}}\;{\text{protein}}\;{\text{content}}\;{\text{(mg/mL)}}\;{\text{in}}\;{\text{cell}}\;{\text{lysate}}}} = {\text{pg/mg}}$$

The criteria for consideration as a skin sensitizer were as follows: SI IL-6 or IL-1α ≥ 3 (Jeon et al. 2019); SI IL-18 ≥ 1.2 (Corsini et al. 2013) in HaCaT assay. SI IL-18 ≥ 2 and SI IL-8 > IL-1α for the epidermal equivalent assay (RHE model) (Gibbs et al. 2013; Galbiati et al. 2018; Coquette et al. 2003). The chemical was considered an irritant in case SI IL-1α > IL-8 (Galbiati et al. 2018; Coquette et al. 2003).

#### Expression levels of inflammatory mediators by RT-qPCR

The expression of genes from inflammatory cytokines (*IL1A*, *IL6*, *IL8* and *TNF*) and long non-coding RNAs involved in inflammation (*NEAT1* and *MALAT1*) was quantified in HaCaT cells exposed to DiPeP 300 nM. For this purpose, HaCaT cells (4 × 10^5^ cells/well—6-well plate) were grown for 24 h in 6-well plates and then were treated for 24 h. RNA was extracted using the Illustra™ RNAspin Mini SV Total RNA Isolation System (Cat. No. 25-0500-71; GE Healthcare), following the manufacturer’s instructions. Extracted RNA was quantified using NanoDrop (Thermo Fisher Scientific), and cDNA was obtained by High-Capacity cDNA Reverse Transcription kit (Cat. No. 4368814; Applied Biosystems). The expression of genes was quantified by quantitative reverse transcription polymerase chain reaction (RT-qPCR) using Power SYBR® Green PCR Master Mix (Cat. No. 4367659; Applied Biosystems). RT-qPCR was performed in technical and biological triplicates. The primer sequences are presented in Supplementary Table S1.

Glyceraldehyde-3-phosphate dehydrogenase (GAPDH) was used as endogenous control, and the expression levels of target genes were normalized relative to control by the 2^−ΔΔCT^ method (Leme et al. 2018). RT-qPCR was performed in technical and biological triplicates (*n* = 3). The differences related to the SC were considered significant when fold-change > 2 and *p* < 0.05.

#### THP-1-based assays for evaluating dendritic cell activation (skin sensitization) and immunomodulatory effects

The h-CLAT assay (OECD TG 442 E) with quantification of IL-8 in THP-1 cells, herein named THP-1 activation assay (Iulini et al. 2022), was used to evaluate KE3 of the AOP for skin sensitization. Briefly, cells were exposed to DiPeP concentrations (0.03–300 nM) for 24 h or 72 h. After exposure, cells were collected into cytometer tubes and centrifuged at 1200 rpm for 5 min. Supernatants were subsequently collected and stored at −20 °C. Cells were marked with FITC mouse anti-human CD86 monoclonal antibody, PE mouse anti-human CD54 monoclonal antibody, before they were incubated for 30 min at 4 °C. Each treatment had κ Isotype control, marked with FITC mouse IgG1 (for CD86) and PE Mouse IgG1 (for CD54 and HLA-DR). Quantification of these membrane markers was assessed by NovoCyte 3000 flow cytometer (ACEA Biosciences, Inc). 10,000 events per treatment were acquired in channels FL-1 (FITC) and FL-3 (PE), and the gate settings strategy was defined according to Iulini et al. (2022). The IL-8 expression in cell supernatant was performed according to the method described in Sect. “ELISA: quantification of cytokines”.

HLA-DR expression in THP-1 cells was determined to elucidate mechanisms related to T-cell activation. Thus, THP-1 cells exposed to DiPeP (30 and 300 nM, across 24 h and 72 h) were incubated with PE mouse anti-human HLA-DR monoclonal antibody for 30 min at 4 °C. PE Mouse IgG1 was used as a κ Isotype control, and this marker was quantified by flow cytometry as described above.

For evaluating the immunomodulatory effects, the LPS-induced THP-1 cell activation was used (Masi et al. 2022). Briefly, THP-1 cells were exposed to DiPeP at 30 and 300 nM for 24 h, before media containing DiPeP (30 or 300 nM) and LPS (lipopolysaccharides from *Escherichia coli* 0127:B8, Sigma, Cat. No. L3129, at 10 ng/mL for CD86 and 1 ng/mL for CD54 assessment) was added for another 24-h treatment. THP-1 cells exposed to DMSO 0.1%-v/v were used as SC. After treatment, cells were centrifuged and labeled with antibodies for CD86 and CD54, as previously described. Supernatants of cells exposed to 10 ng/mL LPS were collected and stored at −20 °C for IL-8 quantification by ELISA (Sect. “ELISA: quantification of cytokines”).

Experiments were performed in triplicate per treatment (technical replicate) and with three batches of cells (biological replicate) (*n* = 3). Flow cytometry data for CD86, CD54, and HLA-DR were determined based on the geometric mean fluorescence intensity (MFI) and the relative fluorescence intensity (RFI) related to control (SC) by the following equation:

$${\text{RFI}} = \frac{{{\text{MFI of chemical treated cells}} - {\text{MFI of chemical treated isotype control cells}}}}{{{\text{MFI of vehicle treated control cells}} - {\text{MFI of vehicle treated isotype control cells}}}}.$$

Positive responses in the THP-1 activation assay were set wherein obtained RFI of CD86 ≥ 1.5 in at least one tested concentration (with cell viability ≥ 50%) or the RFI of CD54 ≥ 2.0 in at least one tested concentration (with cell viability ≥ 50%) and significant increase in IL-8 in any of the tested concentrations (OECD TG 442 E 2022; Mitjans et al. 2008).

#### Statistical analysis

Statistical significance between treated and control cells was determined by Dunnett’s multiple comparison test as part of one-way ANOVA. The results of the RT-qPCR were analyzed by one-way ANOVA, followed by Tukey’s multiple comparisons test using ΔCT values of SC cells and DiPeP-treated cells. Differences were considered significant at p < 0.05.

## Results

### In silico predictions

Results acquired through the adopted in silico tools are summarized in Table 3—both for the parent substances and their primary metabolites. Across each of the five structural profilers, no alerts were matched. It is apparent, therefore, that the assumed DiPeP components bore none of the chemical motifs associated mechanistically with the emergence of skin sensitization—be they related either to protein adduction at KE1, or to protein binding preceding dendritic cell activation at KE3. Each further fell out of the domain of the keratinocyte gene expression rule-set relevant to KE2.

**Table 3:** In silico skin sensitization predictions for DiPeP and metabolites

Compound ID	Structural profiler					QSAR model	
	Skin sensitization reactivity domains	Protein binding, skin sens (GHS)	Protein binding, skin sens (OASIS)	Keratinocyte gene expression	Protein binding, h-CLAT	Skin sensitization (CAESAR)	Skin sensitization (IRFMN/JRC)
	KE1	KE1	KE1	KE2	KE3	KE4	KE4
**1**	No alert matched	No alert matched	No alert matched	Not possible to classify	No alert matched	Non-sensitizer (M)	Sensitizer (L)
**2**						Sensitizer (M)	Sensitizer (L)
**3**						Sensitizer (L)	Non-sensitizer (G)
**4**						Non-sensitizer (G)	Non-sensitizer (G)
**5**						Sensitizer (L)	Non-sensitizer (G)
**6**						Non-sensitizer (G)	Non-sensitizer (G)

(L): low reliability; (M) medium reliability; (G) good reliability

The situation regarding the output of QSAR models, each of which cover events associated with KE4 (T-cell proliferation), was less clear-cut: CAESAR returned sensitizing verdicts for di(2-methylbutyl) phthalate and for each of the two monoesters, whereas IRFMN/JRC judged both parents (but no metabolites) as active. It should be noted that four of out of the five affirmative judgments were labeled by the VEGA software as being of “low reliability”—which stands in contrast to six non-sensitizer returns, each of which were of apparent “good reliability”. This indicates that most of the former sit definitively outside of the applicability domain, and the latter uniformly inside. Accounting for such doubts, and the unanimity of the profiling outcomes, it may be stated that the weight of in silico evidence points toward a lack of sensitization potential for the mixture components (considering likely metabolism).

### In vitro assays

#### HaCaT assay and expression of lncRNAs

**Fig. 2:**
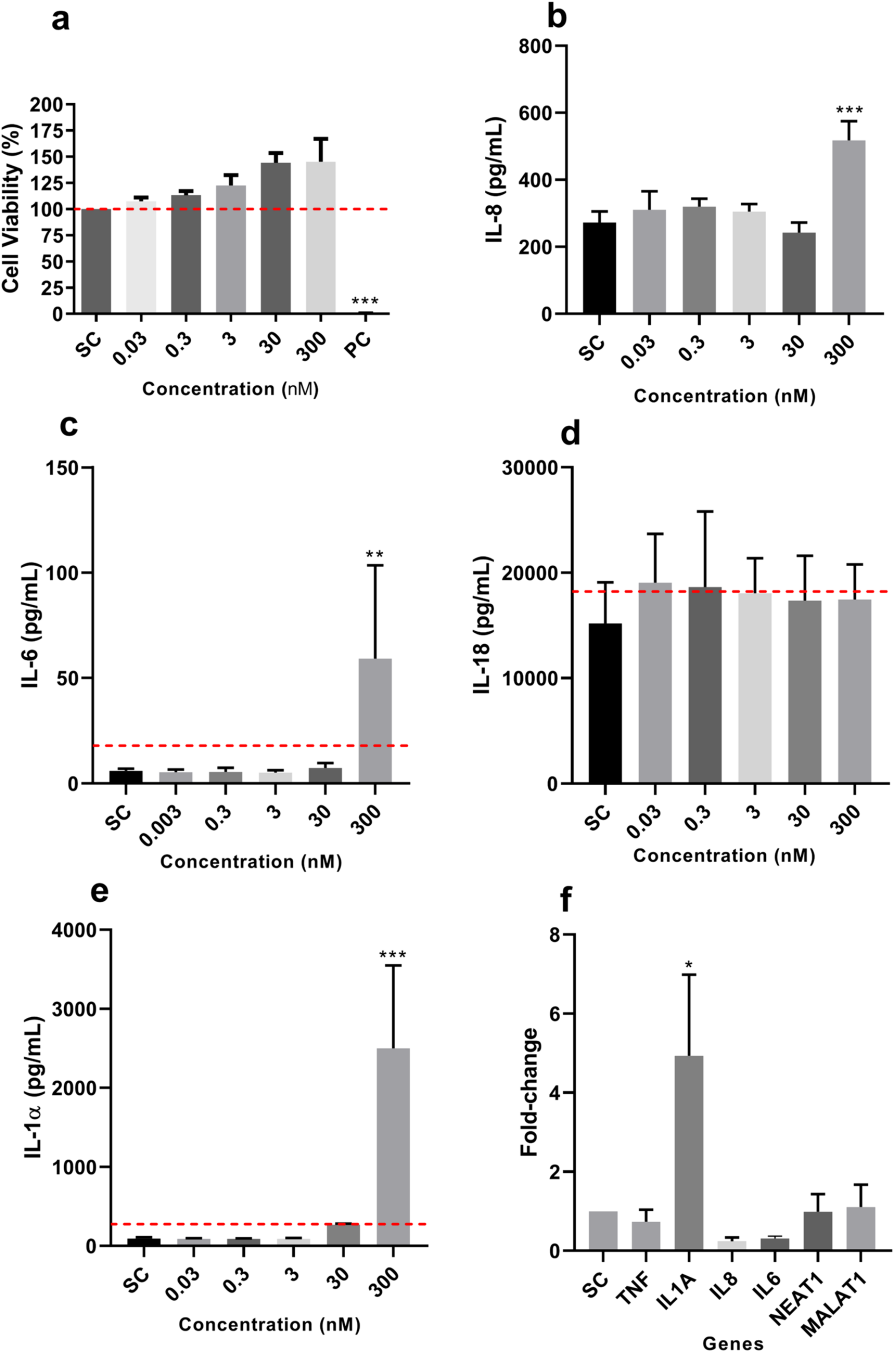
Effect of DiPeP on HaCaT cells after 24 h of exposure. **a** Cell viability. **b–e** Expression of inflammatory cytokines. **f** Gene expression of inflammatory cytokine genes and long non-coding RNAs in HaCaT cells exposed to DiPeP 300 nM. The dotted line in cell viability (Panel **a**) represents the cut-off for cytotoxicity (80% of viability); in the other panels, the dotted line represents the cut-off for being a sensitizer (Panel **b**: SI IL-6 ≥ 3; Panel **c**: SI IL-18 ≥ 1.2; Panel d: SI IL-1α ≥ 3). SC: DMSO 0.1%-v/v; PC: Triton X-100 1%-v/v. SI: stimulation index. Data are expressed as mean ± SD (Panel **a**); as mean ± SEM (Panels **b–f**). Statistical significance between treated and control cells was determined by Dunnett’s multiple comparison test as part of one-way ANOVA; for the RT-qPCR by one-way ANOVA followed by Tukey’s multiple comparisons test using ΔCT values of SC cells and DiPeP-treated cells with *p < 0.05; **p < 0.01; ***p < 0.001

HaCaT cells were exposed to DiPeP at 0.03, 0.3, 3, 30, and 300 nM. Statistically significant cytotoxicity was not observed at any of the tested concentrations, although an increase in proliferation was observed in the highest (Fig. 2a). IL-8 and IL-6 release was significantly increased only at 300 nM; IL-1α and IL-18 intracellular content were quantified, since the release of these cytokines was not observed (data not shown). Intracellular IL-1α expression significantly increased at 30 and 300 nM (Fig. 2b–e), whereas IL-10 release was not detected in HaCaT cells. Significant increase in *IL1A* mRNA level was verified in HaCaT cells exposed to 300 nM DiPeP, while no significant changes in mRNA expression relating either to other tested cytokine genes or to lncRNAs (*NEAT1* and *MALAT1*) were reported (Fig. 2f).

#### Reconstructed human epidermis (RHE model)

**Fig. 3:**
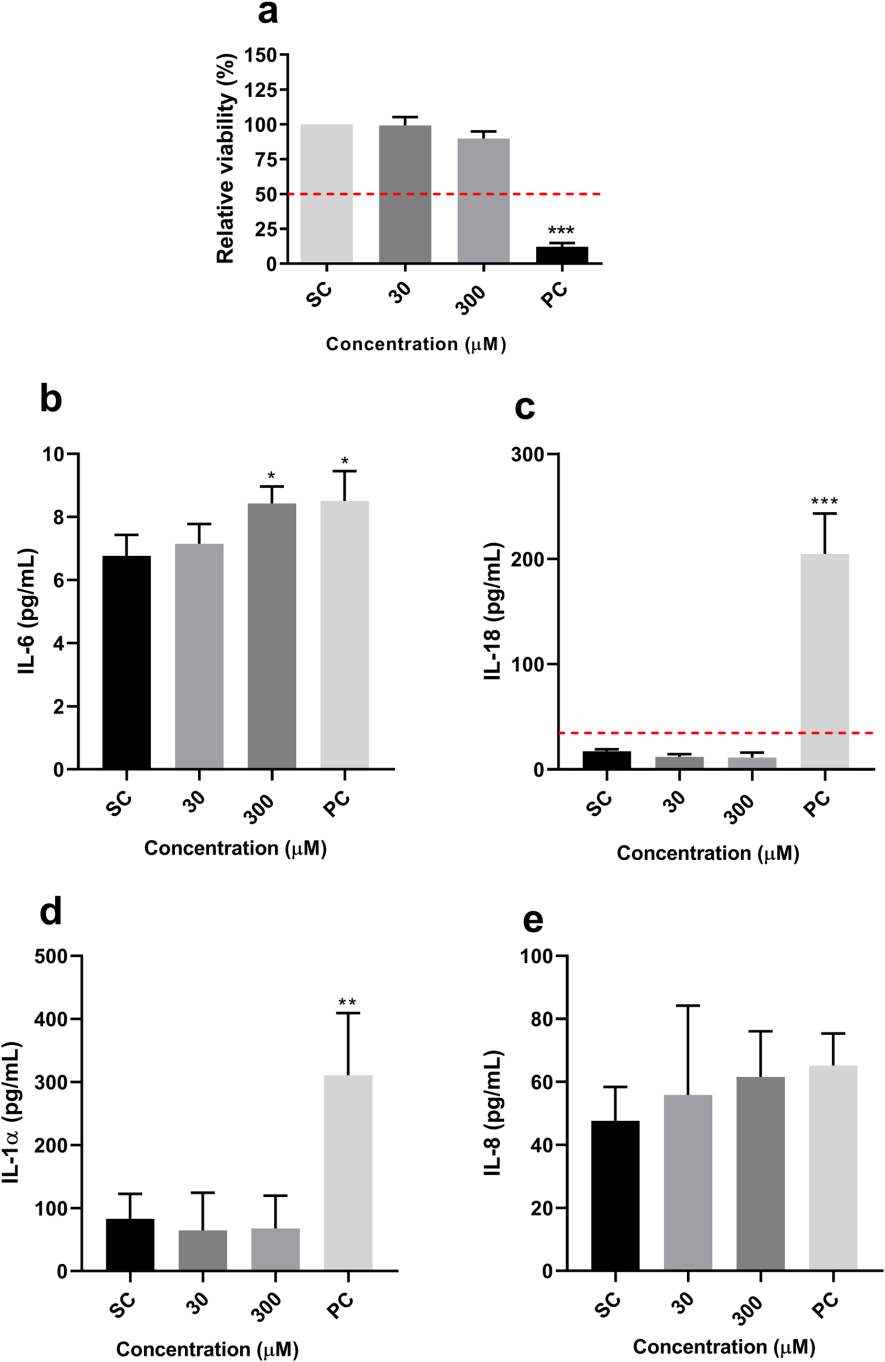
Effect of DiPeP on RHE model after 24 h of exposure.** a** RHE viability. **b-e** Release of pro-inflammatory cytokines. The dotted line in cell viability (Panel **a**) represents the cut-off for cytotoxicity (50% of viability); in Panel **c**, the dotted line represents the cut-off for being a skin sensitizer (SI IL-18 ≥ 2). SC: DMSO 0.1%-v/v; PC: 2,4-dinitrochlorobenzene. SI: fold-change over SC. Data are expressed as mean ± SEM. Statistical significance between treated and control cells was determined by Dunnett’s multiple comparison test as part of one-way ANOVA and unpaired T-test with *p < 0.05; ***p < 0.001

RHE tissues were exposed for 24 h to 30 µM and 300 µM DiPeP dissolved in acetone olive oil (AOO) (4:1). The resulting viability was more than 50% for both the concentrations tested (Fig. 3a), and lower for the PC—which is expected, since this chemical substance can damage the skin tissue due to its inflammatory effect and ability to induce atopic skin lesions (Lian et al. 2022; Bak et al. 2023). Regarding cytokines, a statistically significant increase was verified only for IL-6 (1.24-fold) after exposure to DiPeP at 300 µM (Fig. 3b). All other tested cytokines were not affected (Fig. 3).

#### THP-1 activation assay and LPS-induced THP-1 activation assay

**Fig. 4:**
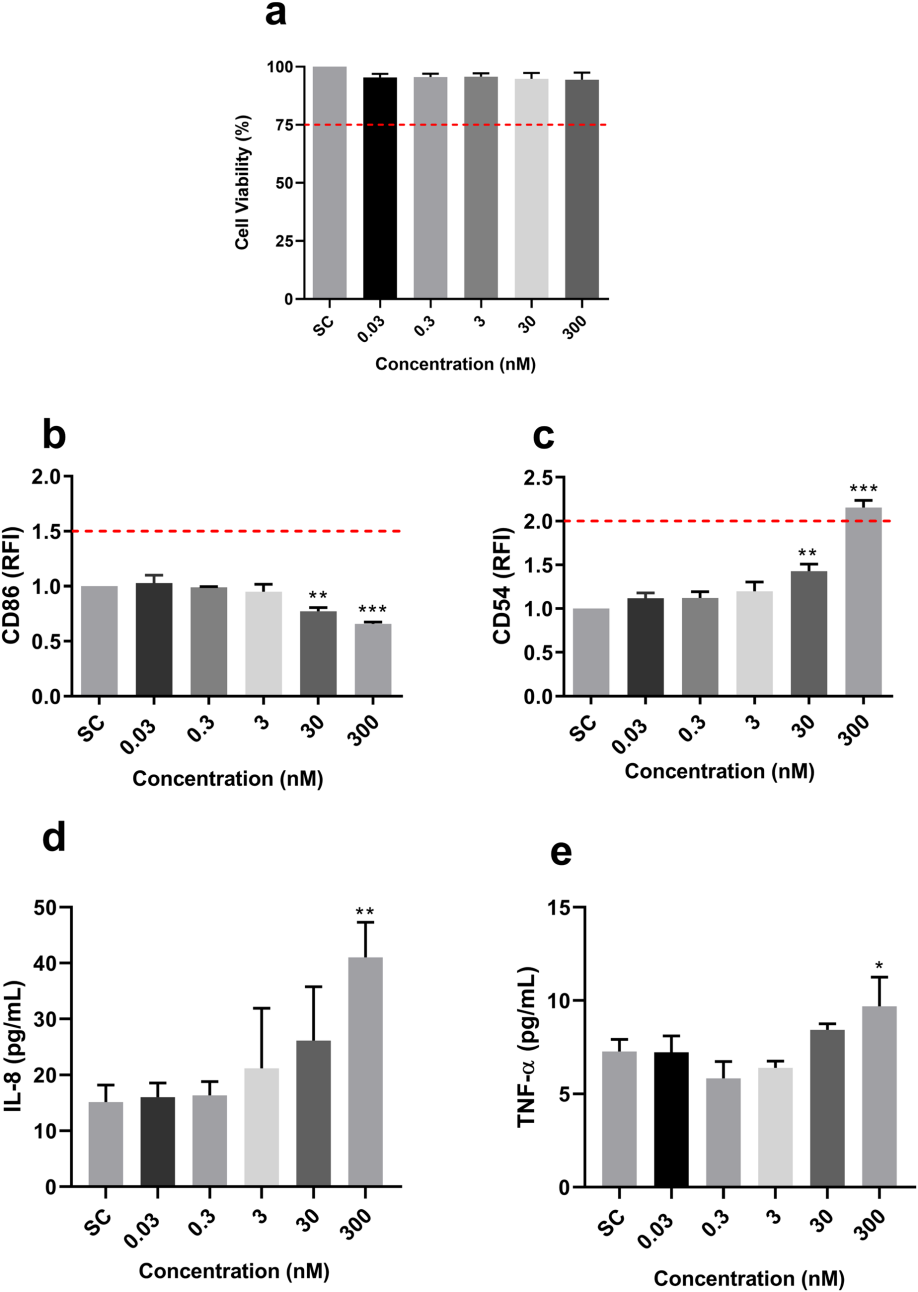
Effect of DiPeP in THP-1 cells after 24 h of exposure.** a** Cell viability; **b–c** Cell surface markers expression; **d–e** Release of pro-inflammatory cytokines. THP-1 cells were exposed to DiPeP (0.03–300 nM). After 24 h, cell viability (Panel **a**), CD54 (Panel **b**) and CD86 (Panel **c**) expression were evaluated through cytometry analysis, while a commercially available ELISA kit evaluated the release of IL-8 (Panel **c**) and TNF-α (Panel **e**). The dotted line is set respectively on 75% for the cytotoxicity evaluation (Panel **a**), and the cut-off for being considered a skin sensitizer by the THP-1 activation assay (Panel **b**: RFI CD86 ≥ 1.5; Panel **c**: RFI CD54 ≥ 2.0). SC: solvent control (DMSO 0.1%-v/v). SI: stimulation index. Statistical significance between treated and control cells was determined by Dunnett’s multiple comparison test as part of one-way ANOVA with *p < 0.05; **p < 0.01; ***p < 0.001. Data are expressed in mean ± SD and represent three independent experiments (*n* = 3)

THP-1 cells were exposed to DiPeP for a period of 24 h, with no significant effect upon viability noted (Fig. 4a). The THP-1 activation assay showed a statistically significant decrease in CD86 expression after 30 and 300 nM DiPeP exposure, and a significant increase for CD54 at these same concentrations (Fig. 4b–c). IL-8 (Fig. 4d) and TNF-α (Fig. 4e) release saw statistically significant increase (2.8-fold and 1.26-fold, respectively) only at 300 nM DiPeP treatment. IL-10 was not detected for any of the tested concentrations.

**Fig. 5:**
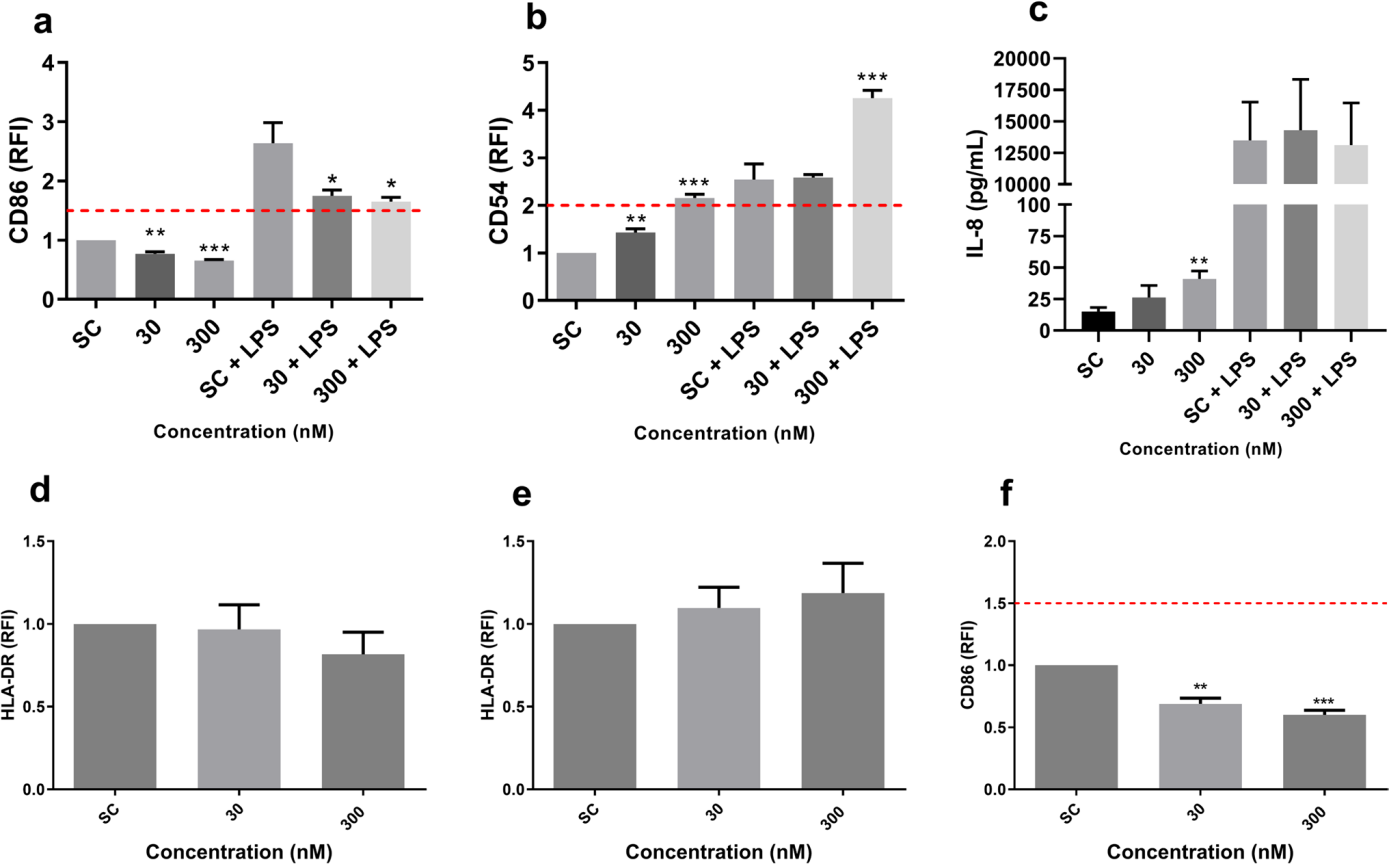
Immunomodulatory effects of DiPeP and HLA-DR expression.** a–b** Cell surface marker expression after 24 h;** c** IL-8 release. **d** HLA-DR expression after 24 h of exposure;** e–f** Cell surface markers expression after 72 h. THP-1 cells were exposed to DiPeP 30 and 300 nM in the presence or absence of LPS (30 nM to CD86 and 3 nM to CD54). After 24 h, CD86 (Panel **a**), CD54 (Panel **b**) and HLA-DR (Panel **d**) expression were evaluated through flow cytometry analysis, while the release of IL-8 (Panel **c**) was assessed by ELISA. HLA-DR (Panel **e**) and CD86 (Panel **f**) expression were also assessed after 72 h. The dotted line is the cut-off for being considered a skin sensitizer by the (Panels **a** and **f**: RFI CD86 ≥ 1.5; Panel **b**: RFI CD54 ≥ 2), according to the h-CLATSC: DMSO 0.1%-v/v. SI: fold-change over SC + LPS for data with LPS and fold-change over SC for data w/o LPS. Data are expressed in mean ± SD. Statistical significance between treated and control cells (SC + LPS or SC) was determined by Dunnett’s multiple comparison test as part of one-way ANOVA with *p < 0.05; **p < 0.01; ***p > 0.001. Data are expressed in mean ± SD and represent three independent experiments (*n* = 3)

The immunomodulatory potential of DiPeP in increasing the inflammatory response was evaluated by LPS-induced THP-1 cell activation assay. DiPeP at 300 nM induced a significant decrease in CD86 expression (Fig. 5a), and a statistically significant increase in CD54 expression (Fig. 5b), within these cells. LPS-induced IL-8 release was not affected by the DiPeP exposure (Fig. 5c).

To elucidate possible mechanisms underlying the reduction in CD86 expression observed in THP-1 cells exposed to DiPeP (with and without LPS induction), expression of the membrane marker HLA-DR was evaluated after 72-h treatment. Differences in HLA-DR expression were not observed under the conditions tested (Fig. 5d–e). Besides 24 h, the expression of CD86 was evaluated at 72 h, with results likewise showing a reduction (thus confirming the trend observed after 24 h) (Fig. 5f).

#### Overall in silico and in vitro data for DiPeP

Table 4 summarizes the main findings regarding the skin sensitization potential (alongside other toxic effects) of DiPeP, obtained by in silico and in vitro methods.

**Table 4:** The overall results for the potential of diisopentyl phthalate (DiPeP) for skin sensitization and immunomodulatory effect by in vitro methods

In vitro: Skin sensitization					
Key events	Model		Endpoint	Result	Conclusion
2: KC activation	HaCaT cells		Release of inflammatory cytokines (IL-6, IL-1α, IL-18, IL-8)	Increased IL-6 and IL-1α release (SI > 3); and IL-8	Sensitizer
	HaCaT cells		Expression of the genes *IL8, IL1A* and *TNF*	Overexpression of *IL1A*	–
	RHE model		Release of inflammatory cytokines (IL-6, IL-1α, IL-18, IL-8)	Slight increase in IL-6 release	Inflammatory agent
3: Activation of DCs	THP-1 cells	THP-1 activation assay based on the h-CLAT (CD86; CD54 expression) and the release of IL-8 Release of TNF-α CD86 expression		CD54 expression RFI CD54 > 1.5); RFI CD54 ≥ 2.0 in at least one tested concentration (300 nM); and significant release of IL-8 Increased release of TNF-α Significant decrease in CD86 expression	Sensitizer Inflammatory agent Immunosuppressor of this marker

Modulation of skin inflammatory responses							
	Model			Endpoint	Results	Conclusion	
	THP-1 cells activated by LPS			Expression of CD86 and CD54, and release of IL-8	Enhances LPS-induced CD54 expression and IL-8 release (SI CD54 > 1.5)	DiPeP may aggravate the immune response generated by inflammatory agents	

*SI* stimulation index

Although results are aligned with single KEs, the endpoints in the assays also recapitulate prior key events

## Discussion

This study employed in silico and in vitro methods to elucidate skin toxicity effects (skin sensitization and immunomodulatory) of the UVCB DiPeP (CAS No. 84777-06-0). For skin sensitization, an approach based on the principles of OECD 497 (OECD 2021) was employed. Protein binding (KE1) was covered by a trio of in silico mechanistic structural profilers, and KC activation (KE2) both in vitro by release of inflammatory cytokines in the HaCaT and RHE models, and in silico by a further profiler indicating potential KeratinoSens assay activity. DC activation (KE3) was assessed by the THP-1 activation assay (modified version of the h-CLAT), which combines measurements of cell surface markers CD54 and CD86 alongside the inflammatory cytokine IL-8—and additionally by an in silico structural profiler associated with the h-CLAT. A pair of statistical QSAR models, trained upon LLNA data, provided linkage to T-cell proliferation (KE4) (Gądarowska et al. 2022; Chayawan et al. 2022).

Outcomes from the various in silico profiling tools showed that neither parent phthalates nor their primary metabolites bore any chemical motifs associated with the emergence of skin sensitization. While the CAESAR and IRFMN/JRC skin sensitization QSARs considered some DiPeP components to be potentially active, such substances lay outside of the respective model applicability domains. Although these negative results were obtained using discrete organic substances (as opposed to the mixture experimentally examined in vitro), the overall in silico outcomes do not support the hypothesis that the compound acts as a sensitizer. Hellwig et al. (1997) found significant differences in developmental and maternal toxicity among a group of phthalate esters, including DiPeP under CAS No. 84777-06-0. The former is dependent upon the binding affinity present between the ester and a specific receptor target—a property which may be influenced significantly by the factors related to the length and branching characteristics of its alkyl chain components (Hlisníková et al. 2020). However, the molecular initiating events underpinning the emergence of such effects are held to differ markedly from those associated with skin sensitization. Skin sensitization arises as a consequence of protein haptenation, itself a product of intrinsic chemical reactivity.

The underlying structural domains associated with tendency to form biomolecule adducts, within the context of this endpoint, are well defined (Enoch et al. 2008)—with neither alkyl phthalates nor their accepted metabolites possessing appropriate molecular features. As such, the minor branching variations distinguishing putative DiPeP isomers shall not be anticipated to have influence upon sensitizing ability. Studies evaluating toxicity among isomers, especially for skin sensitization, are available in the literature. Human patch-test reports indicated that both hydroxypropyl acrylate (a mixture composed of isomers, with CAS No. 25584-83-2), and the functional monomer used in surface coatings (CAS No. 999-61-1), acted as sensitizers to the skin (https://engage.swa.gov.au>widgets>document). Moreover, various menthol forms, such as isomers L-menthol and D-menthol, alongside the racemate and menthol (unspecified isomers), were shown to share similar physicochemical, toxicological, ecotoxicological, and environmental fate properties. Based on their similarities, both the Buehler and LLNA negative results on L-menthol (CAS No. 2216-51-5) could be extended to imply low sensitization potential of all menthol isomers (https://echa.europa.eu/registration-dossier/-/registered-dossier/13758/7/5/2). However, in our case, as no registration dossier was submitted for the substance under CAS No. 84777-06-0, and physicochemical properties are unavailable to proceed for such a rationale. Nevertheless, experimental data under CAS No. 605-50-5 (*i.e.*, Table 1, ID 1) indicated DiPeP as a potential skin sensitizer in an in vivo skin sensitization study (LLNA) (https://echa.europa.eu/registration-dossier/-/registered-dossier/1700/7/5/2).

Although implied as a non-sensitizer by various in silico tools, positive responses in KE2- and KE3-associated in vitro methods performed with DiPeP CAS No. 84777-06-0 were observed. KC activation can be evaluated by the adopted OECD test method (KeratinoSens™ assay), or by methods that have not undergone formal validation, but which nevertheless show good predictivity against the endpoint—such as the HaCaT (Jeon et al. 2019) and RHE assays (Gibbs et al. 2013; Galbiati et al. 2018; Coquette et al. 2003).

In our experiments, HaCaT cells exposed to DiPeP displayed significantly increased IL-8, IL-6, and IL-1α, as well as upregulation of the *IL1A* gene. IL-6 and IL-1α are the best markers to identify sensitizers within the HaCaT assay (Jung et al. 2016; Jeon et al. 2019; Chung et al. 2018; Lee et al. 2015; Mohamadzadeh et al. 1994). Positive response of skin sensitization is defined when IL-6 and/or IL-1α levels are greater than or equal to threefold (SI value), relative to control (Jeon et al. 2019). Thus, the results of the HaCaT assay indicated skin sensitization potential (KE2) for DiPeP (IL-6—8.85-fold increase; IL-1α—22.09-fold increase). In contrast to results found in the HaCaT assay, skin sensitization was not demonstrated in the RHE IL-18 system since the increased release of IL-18 was not observed. For IL-6, this cytokine is a well-known pro-inflammatory cytokine involved in skin inflammation and T-cell differentiation (Kaplanski et al. 2003; Akira et al. 1990; Scheller et al. 2011; Kang et al. 2020). Although RHE models exposed to DiPeP showed significant production of IL-6, this was a slight effect, and further investigation is needed to understand the biological relevance of this effect in inflammatory responses in the skin.

The possible role of DiPeP in inducing KE3 was supported using the THP-1 activation assay, a method that has been adapted with combination of the original h-CLAT and IL-8 quantification, thus providing high sensitivity and accuracy (Mitjans et al. 2008, 2010; Iulini et al. 2022). CD86 and CD54 are co-stimulatory molecules of major histocompatibility complex (MHC) class II, mainly expressed on DCs. CD86 on DCs interacts with CD28 on T cells, which provides T cells with co-stimulatory activation signals (Baravalle et al. 2011). CD54 is associated with activating and priming T cells by strengthening synapses among DCs and T cells (Sheikh et al. 2008). IL-8 is associated with T-cell recruitment, proliferation, and activation (Taub et al. 1996; Kienzl et al. 2019). In this assay, sensitizers are defined as those which demonstrate increased RFI ≥ 1.5 of CD86 or RFI ≥ 2.0 for CD54, and significant expression of IL-8 (Iulini et al. 2022). From the obtained results, DiPeP was positive for skin sensitization (RFI CD54 ≥ 2.0 at 300 nM), and also showed the capacity to activate DCs by significantly increasing IL-8 release, which supports the involvement of DiPeP in KE3. THP-1 cells exposed to DiPeP also showed significant increase in release of TNF-α, a potent inflammatory cytokine capable of stimulating LC activation, motility, and antigen presentation to T cells (Clayton et al. 2017). It was previously reported that TNF-α augments CD54 in a dose-dependent manner without changing CD86 expression in THP-1 cells exposed to skin sensitizers (Miyazawa et al. 2008), thus agreeing with the sole increase in CD54 found in our study.

Overall, the in vitro results demonstrated that DiPeP CAS No. 84777-06-0 exerts a predominant inflammatory effect. However, ultimate findings with respect to skin sensitization potential are inconclusive—with both the HaCaT and THP-1 activation assays returning positive verdicts, and the epidermal equivalent assay registering negative. Of note, despite being positive for KE3 according to the defined criteria within the THP-1 activation assay, an unexpected reduction in CD86 expression was noted in these cells.

Additionally, in contrast to the demonstrated inflammatory and skin sensitization effects of DiPeP in vitro (CAS No. 844777-06-0 in THP-1 activation assay and HaCaT assay), the various in silico tools registered negative verdicts for DiPeP (CAS No. 605-50-5). This contrast is probably related to the composition of UVCB. The predictivity beyond the chemical domains of the individual validation studies remains largely untested. Although new approaches addressing specific aspects of UVCB and difficult-to-test substance assessment are being developed across toxicology, cheminformatics, and regulatory practice, they continue to present a major challenge for risk evaluation (Lai et al. 2022). The issue of testing complex materials has already engaged several groups investigating the extension of the applicability of validated methods of NAMs applicable to a wide range of substances. Kolle and colleagues (2023), comparing the results of 27 difficult-to-test substances (using in vitro and *in chemico* methods) against available in vivo skin sensitization data, observed that these compounds could be out of the applicability domains of validated approaches, so that the value of results should be carefully evaluated.

NAMs, and combinations of the so-called defined approaches reflecting the first three of the four KEs within the skin sensitization AOP, are coming to displace the corresponding in vivo tests—with their predictivity demonstrated to be acceptable (de Souza et al. 2023; Caloni et al. 2022; Wei et al. 2024). However, these predictions are mainly based on the testing of simple substances. “Difficult-to-test” ingredients, including the UVCBs, are placed outside the applicability domains of most in vitro models (Bergal et al. 2020), and are considered extremely challenging to be assessed due to their unknown or variable composition (Kolle et al. 2023).

Besides investigating the skin sensitization potential of DiPeP, this study has also investigated the role of DiPeP in modulating skin inflammation. Results showed that DiPeP can increase LPS-induced CD54 and IL-8 expressions in THP-1 cells, which each demonstrate its immunomodulatory effect in skin inflammation. Surprisingly, DiPeP suppresses CD86 expression in THP-1 cells with and without LPS stimulation. Among the CD86 suppression routes, protein degradation by ubiquitination is the major mechanism controlling surface expression (Baravalle et al. 2011). MARCH1 E3 ubiquitin ligase is responsible for inducing CD86 intracellular degradation via the transmembrane domains, and this mechanism is not associated with CD54 expression (Baravalle et al. 2011; Zhu et al. 2020). Also, MARCH1 leads to MHC-II degradation in the lysosomes (Zhu et al. 2020). Thus, to understand whether the decreased CD86 expression in DiPeP treatment is influenced by MARCH1, the expression of HLA-DR (MHC-II molecule) was evaluated. Interestingly, HLA-DR expression was not affected by DiPeP exposure at two time points tested (24 h and 72 h), suggesting that MARCH1 is not related to this effect; however, additional studies shall thus be required to elucidate the mechanisms behind this. Anyhow, the reduced CD86 expression cannot be considered as an anti-inflammatory effect of DiPeP, since the expression of other inflammatory markers in both KC-based assay and THP1 cells occurred at significant levels.

The potential of DiPeP to modulate epigenetic markers was another endpoint investigated, since transient changes in DNA can influence the pathophysiology and severity of inflammatory skin diseases (Möbus et al. 2020). Thus, two highly expressed lncRNAs, a class of molecules greater than 200 nucleotides (nt) in length) occurring in all skin cell types (Shefler et al. 2022) were selected for this purpose. MALAT1 (metastasis-associated lung adenocarcinoma transcript 1) and NEAT1 (nuclear-enriched abundant transcript 1) often form relatively stable secondary and higher structures, may regulate various cell signaling molecules (Tang et al. 2020) and have been associated with psoriasis, for instance (Zhang et al. 2021). A recent study from our group demonstrated that the *MALAT1* and *NEAT1* genes can have their expression affected by the conventional chemical substance Octylphenol (de Souza et al. 2023). However, unlike in the study of Octylphenol, DiPeP did not change the expression of these lncRNA genes by either downregulation or upregulation of their expression.

## Conclusion

Overall, the in silico and in vitro data described herein were not capable of providing a clear response regarding the skin sensitization potential of the UVCB DiPeP (CAS No. 84777-06-0). Thus, the substance was reported as inconclusive for skin sensitization—contrasting with discrete DiPeP (CAS No. 605-50-5), which is classified as skin sensitizer by LLNA assay (animal-based method). Considering that UVCB substances could fall outside the applicability domain of the in silico and/or in vitro assays, inconclusive predictions by NAMs are not uncommon. Further studies are needed to elucidate the skin sensitization potential of the UVCB DiPeP, as well as to better overcome potential limitations of non-animal methods in evaluating the skin sensitization of substances such as UVCBs. Despite the inconclusive evaluation for UVCB DiPeP skin sensitization, this substance showed an inflammatory effect in the 3D skin model (increased IL-6 release) and presented a clear immunomodulatory effect related to DC activation.

### Supplementary Information

Below is the link to the electronic supplementary material.

Supplementary file1 (DOCX 15 KB)

## Data Availability

Data will be available on request.
