# Supplementary material for: The evaluation of skin sensitization potential of the UVCB substance diisopentyl phthalate by in silico and in vitro methods
Source: Arch Toxicol. 2024 May 28;98(7):2153–71. doi: 10.1007/s00204-024-03738-x (PMC11169023; doi:10.1007/s00204-024-03738-x)
Supplement: Supplementary file 1 — Supplementary file1 (DOCX 15 KB) [file 204_2024_3738_MOESM1_ESM.docx]

Table S1. Primer sequences used in the RT-qPCR.

| Genes | Direction | Sequence |
| --- | --- | --- |
| *GAPDH* | F | 5′-ACCCACTCCTCCACCTTTGA-3′ |
|  | R | 5′-CTGTTGCTGTAGCCAAATTCGT-3′ |
| *TNF* | F | 5'-CCGAGGCAGTCAGATCATCTT-3' |
|  | R | 5'-AGCTGCCCCTCAGCTTGA-3' |
| *IL6* | F | 5′-AGATTTGAGAGTAGTGAGGAACAAG-3′ |
|  | R | 5′-CTGGCATTTGTGGTTGGGTC-3′ |
| *IL8* | F | 5′-ATACTCCAAACCTTTCCACCCC-3′ |
|  | R | 5′-CCTCTGCACCCAGTTTTCCTTG-3′ |
| *IL1A* | F | 5′-CAGCCAGAGAGGGAGTCATT-3′ |
|  | R | 5′-GGAGGAACTGTCTTCTTCATTTTCA-3′ |
| *NEAT1* | F | 5’-TCTCCATTTCCCCATCTGAG-3' |
|  | R | 5’-CAGCCACAGAAAAGGGAGAG-3’ |
| *MALAT1* | F | 5’-GGGTGTTTACGTAGACCAGAACC-3’ |
|  | R | 5’-CTTCCAAAAGCCTTCTGCCTTAG-3' |

F: forward; R: reverse.
